# Two-Stage Framework for Faster Semantic Segmentation

**DOI:** 10.3390/s23063092

**Published:** 2023-03-14

**Authors:** Ricardo Cruz, Diana Teixeira e Silva, Tiago Gonçalves, Diogo Carneiro, Jaime S. Cardoso

**Affiliations:** 1 Faculty of Engineering, University of Porto, 4200-465 Porto, Portugal; rpcruz@fe.up.pt (R.C.); up201805131@edu.fe.up.pt (D.T.e.S.); tiago.f.goncalves@inesctec.pt (T.G.); 2 INESC TEC—Institute for Systems and Computer Engineering, Technology and Science, 4200-465 Porto, Portugal; 3 Bosch Car Multimedia, 4705-820 Braga, Portugal; diogo.carneiro@pt.bosch.com

**Keywords:** semantic segmentation, deep learning, computer vision

## Abstract

Semantic segmentation consists of classifying each pixel according to a set of classes. Conventional models spend as much effort classifying easy-to-segment pixels as they do classifying hard-to-segment pixels. This is inefficient, especially when deploying to situations with computational constraints. In this work, we propose a framework wherein the model first produces a rough segmentation of the image, and then patches of the image estimated as hard to segment are refined. The framework is evaluated in four datasets (autonomous driving and biomedical), across four state-of-the-art architectures. Our method accelerates inference time by four, with additional gains for training time, at the cost of some output quality.

## 1. Introduction

Neural networks for segmentation typically produce a probability, for each pixel, of belonging to the region of interest [1,2,3,4]. The same number of arithmetic operations is performed for all pixels, which seems computationally inefficient because there are regions of the image that may be harder to segment than others. Intuitively, that does not seem how humans would produce a manual segmentation. We would create a rough draft and then refine the parts of the segmentation that require more detail. Furthermore, many segmentation applications are imbalanced. The background predominates, and the background is often easier to segment.

In applications with very high-resolution images, such as high-resolution digital microscopes, a common practice is to split the image into patches and process each patch separately [5]. However, such an approach does not solve the fact that resources are being spent equitably when it makes sense to unevenly deploy these resources across the regions of the images. Our proposal tries to be more selective on the patches it chooses. Other areas that may also benefit from low-cost segmentation, even with some accuracy penalty, include single-board computers, such as the Raspberry Pi microcontroller that are used for smart houses, security, or in retail to estimate customers entering a store [6].

**Figure 1:**
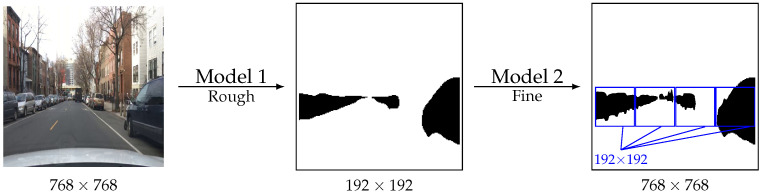
Illustration of the proposal (BDD dataset).

As illustrated by Figure 1, the proposal is to produce a sequential segmentation method whereby Model 1 segments a lower-resolution version of the image. Based on the probability scores from this first model, the harder-to-segment regions are identified. These regions are then fed into Model 2 for further processing. Finally, the output of both models is then combined.

Iterative segmentation methods already exist [7,8,9,10], but their focus is on improving the quality of the segmentation, not the speed. Our proposal is applicable to every type of high-resolution image, but it is tested over two types of images (biomedical and autonomous driving).

The paper expands on a previous conference paper [11] by introducing three crucial improvements to the pipeline and an expansion of the experiments: (i) the second model is trained from the first model; (ii) the first model is connected to the second model to provide context; and (iii) three sampling strategies are considered to choose the image patches for the training of the second model.

In addition to this introduction, the paper is organized as follows: Section 2 better explores the related work, Section 3 explains the proposed algorithm, Section 4 details the experiments performed and discusses the results, and finally Section 5 concludes the paper. The source code with the implementation used to produce the experiments of this paper is publicly available at https://github.com/rpmcruz/faster-segmentation (accessed on 12 February 2023).

## 2. Related Work

**Figure 2:**
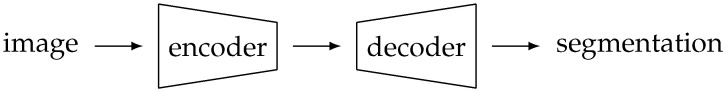
Generic segmentation network.

In broad strokes, deep learning architectures for neural networks consist of two encoder–decoder sequential blocks, as shown in the following Figure 2.

The encoding phase reduces the image into a smaller and more compact, higher-level representation, while the decoder projects that latent representation into a segmentation with the same size as the original image. Four major architectures are considered in this work: fully convolutional network (FCN) [1], SegNet [2], U-Net [3], and DeepLab [4]. FCN is considered a pioneering approach to deep-based image segmentation. It uses successive convolutions for the encoder, but a single dense layer for the decoder that is then reshaped to the final shape [1]. SegNet also uses successive convolutions for the decoder [2]. U-Net introduces extra “skip connections”, which consist in concatenating the activation map produced by each encoder layer to the corresponding decoder layer; this improves gradient fluidity and, typically, the output quality [3]. DeepLab tries to improve the checkerboard effect, sometimes found on segmentation methods, by diluting the distinction between the encoder–decoder through the application of atrous convolutions that avoid the need to successively reduce the input image by instead enlarging the convolution kernels [4].

As shown in Table 1, as the input doubles, the number of floating point operations (FLOPs) quadruples. The memory required also increases. This makes applying deep-based models to high-resolution images computationally expensive. In areas such as computational pathology, one of the main limitations is related to the large file size due to the high-resolution (and different magnifications) of the whole slide images. Patch-based methods are therefore common. In these approaches, images are divided into several, smaller patches that are possible for neural networks to process [5].

There is work that focuses on improving training and/or inference time, and it typically involves working with multiple scales, but not on semantic segmentation. Google AI performs alpha matting on mobile devices (i.e., extracting a foreground object) by a two-stage process, due to computational constraints, whereby a neural network performs an initial step, and a secondary network is used only on areas that might require further work [12]. Such an approach has been adapted to depth estimation [13]. For the purpose of image classification, ref. [14] has multiple inputs of varying scales, which are decided by attention mechanisms. Another approach that has been used for classification and object detection is to choose these patches recurrently by reinforcement learning [15,16].

## 3. Method

Conventional methods apply the computational effort uniformly across the input space, which seems inefficient. Therefore, our proposal consists of the following steps, which are made clearer by the pseudocode in Algorithm 1:

Step 1. Model 1 segments a low-resolution version of the image (line 1);
Step 2. the poorly segmented image patches are identified based on the probabilities produced by Model 1 (line 2); and
Step 3. Model 2 refines these patches (line 3).

**Algorithm 1:** Pseudocode of the proposed method.**Input:** two models, $f^{\left(1\right)}$ and $f^{\left(2\right)}$, and an image input *x*1: $\hat{p}\leftarrow s_{↑}\left(f^{\left(1\right)}\left(s_{↓}\left(x\right)\right)\right)$2: $\mathrm{patches}\leftarrow \{\left(i,j\right)\;|\;g\left(h\left(c_{i,j}\left(\hat{p}\right)\right)\right)\}$3: $c_{i,j}\left(\hat{p}\right)\leftarrow f^{\left(2\right)}\left(c_{i,j}\left(x\right)\right)$,    ∀i,j∈patches.where $s_{↑}$ and $s_{↓}$ are upscale and downscale interpolations, $c_{i,j}$ crops the (i,j) patch,*g* is the selection function (Section 3.1), function *h* produces an uncertainty scorefor a patch by calculating the average of the uncertainty associated with theprobability of each pixel, $u\left(p\right)=-p\log_{2}p$, so that highly uncertain regionscorrespond to those with probabilities closest to 0.5.

**Figure 3:**
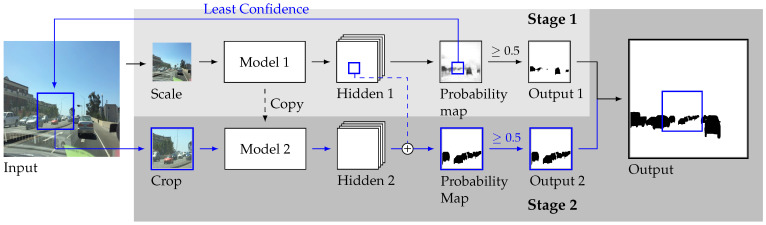
Two-stage segmentation.

Notice that both models use the same architecture and receive the same image size, except that Model 1 is a scaled-down version of the image, while Model 2 is a crop of the image. For example, if Model 1 receives one image downscaled by 4×, then Model 2 receives images cropped in 4×4 from the original so that the input shape of both models is the same. The full pipeline is illustrated in Figure 3.

### 3.1. Selection Method

At each epoch, while the model is being trained, one patch of each image is sampled by *g* by using one of the following strategies.

1. Random: uniform sampling.
2. Weighted: Sampling is weighted by the uncertainty produced by Model 1. Shannon entropy is used as a measure of uncertainty by taking the probability map *p* produced by Model 1, and computing an uncertainty *h* score. This uncertainty is then normalized and used as the sampling probability.
3. Highest: The highest uncertainty patch is always selected. While this seems to be the most obvious approach, it also removes some stochasticity and variability from the training of Model 2.

The three strategies are experimented with in Section 4.3. After the model has been trained, a threshold is used to select the patches with uncertainty above a certain threshold (typically 0.5).

### 3.2. Extension

This paper is an expansion from our conference paper [11] and introduces three crucial improvements on the pipeline and expands the experiments: (1) transfer learning is used so that Model 2 is trained on top of Model 1 (black dashed lines in the figure); (2) Model 1 provides context to Model 2 by elementwise addition between the penultimate layer of both models (blue dashed lines); (3) during training for step 2, one patch of each image is selected for Model 2 by using one of three sampling strategies.

## 4. Experiments and Results

### 4.1. Datasets

Four datasets were used: two from the autonomous driving literature (BDD [18] and KITTI [19]), and two from the biomedical literature (BOWL [20] and PH2 [21]), all of which are detailed in Table 2. The biomedical datasets consist of binary classification (BOWL for cell recognition, while PH2 is skin lesion recognition), and the autonomous driving datasets were also used for binary classification (to recognize vehicles). The train–test split was 70-30, except for BDD, which already comes partitioned by the authors.

### 4.2. Experimental Setup

The four semantic segmentation models from the literature discussed in Section 2 are experimented with: FCN [1], SegNet [2], U-Net [3], and DeepLab v3 [4]. All these models share the same backbone (ResNet-50 [17]), which is pretrained in ImageNet [22]. The implementations that came with Torchvision [23] are used for FCN and DeepLab v3, while SegNet and U-Net are implemented by us, also making use of the backbone ResNet-50 from Torchvision.

As typically done in segmentation tasks, focal loss is used for training [24], while the evaluation metric is the Dice coefficient, the segmentation equivalent of the F_1_-score used for classification, which takes class imbalance into account. The optimizer is Adam with a learning rate of $10^{-4}$ that is trained for 20 epochs.

For data augmentation of the aforementioned datasets, the transformations are horizontal flipping, jittering of brightness and contrast by 0.1, and a random translation shift of 10% the size of the image performed by an upscale followed by a crop. All images are resized to 768×768.

### 4.3. Results

The main results are presented in Table 3. The four architectures are contrasted and evaluated in the four datasets. The baseline (using a single model in the entire image) is contrasted against the pipeline (using 16 patches, weighted sampling, and an uncertainty threshold of 0.25). The quality of the output is measured by the Dice metric, while time latency is provided for both training (in GPU) and inference (in CPU).

The proposal presents considerable gains in latency time—on average, training time is reduced by 42.0%, and inference time by 74.7%—at the cost of slightly reducing the quality of the segmentation maps produced by 6.3%, on average.

The italicized values in the table will now be subject to four ablation studies.

### 4.4. Ablation Studies

In Table 4, we attempt to estimate the effect of changing several aspects of the pipeline. The architecture has been fixed as DeepLab, and the other parameters are changed. Not surprisingly, some columns predominate (almost fully bold) for reasons now discussed.

1. The number of patches used to divide the image: There is a general reduction of quality as the number of patches is increased. However, it should be noted that this comes with a gain in latency because the more patches used, the smaller the input sizes, which means lower FLOPs, as previously detailed in Table 1.
2. The sampling strategy used to select the patches during the training of Model 2: The results of varying the way that patches are selected during the training of Model 2 are based on the uncertainty produced by Model 1. The differences are not considerable, albeit always choosing the highest uncertainty patch or sampling weighted by the normalized uncertainty seem like the best strategies.
3. The impact of changing the uncertainty threshold with which patches are selected for Model 2 during inference: The threshold chooses the patches from Model 1 to be refined by Model 2. The lower the uncertainty threshold, the more patches will be selected. Clearly, the more patches that are refined by Model 2, the better the final segmentation, naturally at a proportional time cost.
4. Whether certain additional features from the proposal are relevant: The dashed lines illustrated in the pipeline from Figure 3 are disabled depending on whether Model 1 is used to pretrain Model 2 (fine tune) or whether an activation map is given from Model 1 to Model 2 (context). In both cases, both of these aspects of the pipeline clearly aid in improving the output quality, because disabling them lowers quality.

## 5. Conclusions

It seems wasteful for the semantic segmentation model to perform the same computational effort across the pixel space of the image. The current work proposes a two-stage pipeline whereby a rough segmentation is produced by a first model using a low-resolution image, followed by another model that refines the most intricate regions of the image by using patches of the original high-resolution image. These regions are selected based on uncertainty estimates produced by the first model. In the end, both outputs are combined to form the final segmentation.

The approach is validated on four datasets and four architectures. While the proposal reduces output quality by 6%, it reduces training time by 42% and inference time by 75%. This shows that the proposal may show a path for considerable efficiency gains, while work is still required to bridge the gap in terms of output quality. The large gains in efficiency may justify the accuracy penalty for certain applications, such as when using resource-limited microcontrollers for noncritical applications.

In this work, the image is divided into uniformly distributed contiguous patches. In future work, it would be interesting to allow more flexibility in how the image is divided into patches to avoid cases in which an object is cut in half between patches. Furthermore, training both models from end to end would have been desirable, possibly with an attention mechanism.

## Figures and Tables

**Table 1:** Number of parameters and FLOPs per architecture.

		FLOPs ($10^{6}$)				
Architecture	#Params ($10^{6}$)	96×96	192×192	192×192	384×384	768×768
ResNet-50 *	23.5	215	759	3035	12,142	48,567
DeepLab v3 [4]	42.0	1533	6132	24,527	98,107	392,425
U-Net [3]	73.4	695	2413	9653	38,613	154,452
SegNet [2]	48.4	594	1776	7105	28,421	113,684
FCN [1]	35.3	1316	5265	21,060	84,238	336,952

* All our models use this ResNet-50 [17] as the encoder backbone.

**Table 2:** Datasets for semantic segmentation.

Dataset	Category	N	Avg Res	% Fg	Example
BDD [18]	Autonomous driving	8000	720×1280	9.7	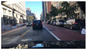
KITTI [19]	Autonomous driving	200	375×1271	6.6	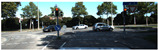
BOWL [20]	Biomedical	670	328×369	13.5	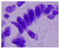
PH2 [21]	Biomedical	200	575×766	31.8	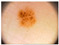

**Table 3:** Results across the several architectures. The proposal presents considerable gains in latency time at the cost of some quality of the segmentation output (best results in **bold**). The *italic* values will be subject to the subsequent ablation study.

		Baseline			Proposal		
Architecture	Dataset		Time (s)			Time (s)	
		Dice (%)	Train	Inference	Dice (%)	Train	Inference
Average		**87.6**	181.0	5407.0	82.1	**105.0**	**1369.9**
DeepLab	BDD	**86.4**	661.7	23,191.8	*79.8*	**227.0**	**5416.6**
	KITTI	**92.4**	15.9	1363.7	*85.6*	**11.5**	**257.5**
	BOWL	**87.0**	94.0	4622.2	*81.7*	**81.2**	**1772.0**
	PH2	91.9	15.8	1384.3	***94.5***	**11.0**	**544.9**
U-Net	BDD	**82.7**	536.8	11,166.0	81.2	**356.7**	**2338.7**
	KITTI	**88.5**	13.3	687.7	72.6	**10.8**	**133.4**
	BOWL	82.9	93.3	2201.5	**83.2**	**79.7**	**966.7**
	PH2	90.5	13.0	682.3	**91.1**	**10.4**	**251.6**
SegNet	BDD	**85.9**	529.9	9090.0	76.9	**354.4**	**2287.3**
	KITTI	**81.2**	13.3	549.9	71.1	**10.7**	**203.9**
	BOWL	**78.9**	91.1	1868.9	67.1	**79.1**	**1185.1**
	PH2	**92.1**	13.0	538.5	89.5	**10.3**	**266.1**
FCN	BDD	**86.1**	679.2	21,977.2	79.9	**335.5**	**3902.6**
	KITTI	**92.9**	16.3	1336.0	84.7	**10.8**	**219.9**
	BOWL	**87.0**	93.2	4507.4	79.4	**80.9**	**1633.9**
	PH2	94.7	16.1	1343.8	**94.8**	**10.3**	**538.0**

**Table 4:** Ablation studies for several parts of the proposed method (best results in bold). DeepLab is used as the architecture.

	(1) Dice (%) per #patches				
**Dataset**	**4**	**16 ${}^{†}$**	**64**	**128**	
BDD	**84.4**	79.8	80.1	68.1	
KITTI	**89.3**	85.6	80.3	53.4	
BOWL	**85.5**	81.7	78.9	75.1	
PH2	**91.5**	94.5	93.1	88.6	
	**(2) Dice (%) per patch selection**				
**Dataset**	**Random**		**Weighted ${}^{†}$**		**Highest**
BDD	79.3		79.8		**79.9**
KITTI	84.4		85.6		**86.1**
BOWL	80.7		**81.7**		81.4
PH2	93.9		94.5		**94.6**
	**(3) Dice (%) per uncertainty threshold**				
**Dataset**	**≥1**	**≥0.75**	**≥0.5**	**≥0.25 ${}^{†}$**	**≥0**
BDD	75.3	75.5	76.0	79.8	**84.4**
KITTI	80.4	80.4	80.7	85.6	**90.4**
BOWL	76.5	76.7	78.0	81.7	**87.2**
PH2	93.9	93.9	94.0	**94.5**	94.4
	**(4) Dice (%) per disabled feature**				
**Dataset**	**Disable fine tune**		**Disable context**		**Full-featured ${}^{†}$**
BDD	77.1		79.0		**79.8**
KITTI	80.2		82.2		**85.6**
BOWL	80.4		78.9		**81.7**
PH2	94.2		94.2		**94.5**

^†^ This column uses the same parameters as in the previous experiments (italicized values in Table 3).

## References

[1] Long J., Shelhamer E., Darrell T. Fully convolutional networks for semantic segmentation. Proceedings of the IEEE Conference on Computer Vision and Pattern Recognition (CVPR).

[2] Badrinarayanan V., Kendall A., Cipolla R. (2017). SegNet: A deep convolutional encoder-decoder architecture for image segmentation. IEEE Trans. Pattern Anal. Mach. Intell..

[3] Ronneberger O., Fischer P., Brox T. (2015). U-Net: Convolutional networks for biomedical image segmentation. Proceedings of the International Conference on Medical Image Computing and Computer-Assisted Intervention (MICCAI).

[4] Chen L.C., Papandreou G., Schroff F., Adam H. (2017). Rethinking atrous convolution for semantic image segmentation. arXiv.

[5] Wang C., Zhao Z., Ren Q., Xu Y., Yu Y. (2019). Dense U-Net based on patch-based learning for retinal vessel segmentation. Entropy.

[6] Kondaveeti H.K., Bandi D., Mathe S.E., Vappangi S., Subramanian M. A review of image processing applications based on Raspberry-Pi. Proceedings of the 2022 8th International Conference on Advanced Computing and Communication Systems (ICACCS).

[7] Fernandes K., Cruz R., Cardoso J.S. Deep image segmentation by quality inference. Proceedings of the 2018 International Joint Conference on Neural Networks (IJCNN).

[8] Kim J.U., Kim H.G., Ro Y.M. Iterative deep convolutional encoder-decoder network for medical image segmentation. Proceedings of the 2017 39th Annual International Conference of the IEEE Engineering in Medicine and Biology Society (EMBC).

[9] Wang W., Yu K., Hugonot J., Fua P., Salzmann M. Recurrent U-Net for resource-constrained segmentation. Proceedings of the IEEE/CVF International Conference on Computer Vision (ICCV).

[10] Banino A., Balaguer J., Blundell C. PonderNet: Learning to Ponder. Proceedings of the 8th ICML Workshop on Automated Machine Learning (AutoML).

[11] Silva D.T., Cruz R., Gonçalves T., Carneiro D. Two-stage Semantic Segmentation in Neural Networks. Proceedings of the Fifteenth International Conference on Machine Vision (ICMV 2022).

[12] Google AI Blog (2022). Accurate Alpha Matting for Portrait Mode Selfies on Pixel 6. https://ai.googleblog.com/2022/01/accurate-alpha-matting-for-portrait.html.

[13] Miangoleh S.M.H., Dille S., Mai L., Paris S., Aksoy Y. Boosting monocular depth estimation models to high-resolution via content-adaptive multi-resolution merging. Proceedings of the IEEE/CVF Conference on Computer Vision and Pattern Recognition (CVPR).

[14] Yu Q., Wang H., Kim D., Qiao S., Collins M., Zhu Y., Adam H., Yuille A., Chen L.C. CMT-DeepLab: Clustering Mask Transformers for Panoptic Segmentation. Proceedings of the IEEE/CVF Conference on Computer Vision and Pattern Recognition (CVPR).

[15] Mnih V., Heess N., Graves A. Recurrent models of visual attention. Proceedings of the 27th International Conference on Neural Information Processing Systems.

[16] Ba J., Mnih V., Kavukcuoglu K. Multiple object recognition with visual attention. Proceedings of the International Conference on Learning Representations.

[17] He K., Zhang X., Ren S., Sun J. Deep residual learning for image recognition. Proceedings of the IEEE Conference on Computer Vision and Pattern Recognition.

[18] Yu F., Chen H., Wang X., Xian W., Chen Y., Liu F., Madhavan V., Darrell T. BDD100K: A Diverse Driving Dataset for Heterogeneous Multitask Learning. Proceedings of the 2020 IEEE/CVF Conference on Computer Vision and Pattern Recognition (CVPR).

[19] Alhaija H., Mustikovela S., Mescheder L., Geiger A., Rother C. (2018). Augmented Reality Meets Computer Vision: Efficient Data Generation for Urban Driving Scenes. Int. J. Comput. Vis. (IJCV).

[20] Kaggle (2018). 2018 Data Science Bowl. https://www.kaggle.com/c/data-science-bowl-2018.

[21] Mendonça T., Ferreira P.M., Marques J.S., Marcal A.R., Rozeira J. PH^2^–A dermoscopic image database for research and benchmarking. Proceedings of the 2013 35th Annual International Conference of the IEEE Engineering in Medicine and Biology Society (EMBC).

[22] Russakovsky O., Deng J., Su H., Krause J., Satheesh S., Ma S., Huang Z., Karpathy A., Khosla A., Bernstein M. (2015). ImageNet Large Scale Visual Recognition Challenge. Int. J. Comput. Vis. (IJCV).

[23] Marcel S., Rodriguez Y. Torchvision the machine-vision package of torch. Proceedings of the 18th ACM International Conference on Multimedia.

[24] Lin T.Y., Goyal P., Girshick R., He K., Dollár P. Focal loss for dense object detection. Proceedings of the IEEE International Conference on Computer Vision (ICCV).

